# Observations on Black Vomit

**Published:** 1828-02

**Authors:** William Lyons

**Affiliations:** Staff Surgeon.


### BLACK VOMIT.

*Observations on Black Vomit.* By William Lyons, Staff Surgeon.

From my situation of principal medical officer in the island of Dominica, West Indies, and from the fatality of the fever of that island, my attention was given in a particular manner to the consideration of its phenomena. Black vomit, one of its most alarming symptoms, soon fell under consideration; and although I was soon enabled to convince myself of its identity with altered blood, from an attentive examination of that fluid when ejected from the stomach, and the state and contents of the stomach after death, and likewise from an examination of the liver and tubili biliferi, yet the hitherto inexplicable mutation of the blood's colour to black remained in its concealment.

The black colouring matter of this fluid was separated by filtration in the easiest manner, and its powerful acid qualities were proved by its active effervescence with alkalies, as well as by its action on the enamel of the patients' teeth, and by its powerfully ascescent smell: but it was not until I observed, in the examination of recruits at this station, that an immense quantity of muriatic acid is constantly secreted from the skin, with the insensible perspiration, that I brought to my mind Dr. Prout's discovery of this acid's being the principal one secreted in morbid conditions of the stomach, and with it the idea of ascertaining the effects of this acid upon the blood.

Having made the experiment of adding muriatic acid to blood, the colour of the blood was instantly altered to a deep black, and, when diluted with water, presented a liquid which I should have declared, from mere inspection, to be black vomit. This experiment has been tried by Dr. Hope, at my suggestion, and the results have been the same, even when the muriatic acid had been so far reduced with water as to be hardly capable of turning the edges of the teeth; and is supposed by Dr. Hope to be an interesting adaptation of Dr. Prout's discovery to the explanation of the symptom of black vomit.

I am persuaded that if these experiments, or rather facts, shall turn out to be a satisfactory manner of accounting for black vomit, that it will afford you pleasure ; and the more so as the same facts may be fairly brought in explanation of malaria, &c. : it will also be an additional argument of the futility of urging that the black vomit is a symptom which predicates the contagious nature of the fever in which it is observed.

Whilst upon this subject, it may not be amiss to mention the successful treatment of a case of fever with black vomit, by the use of nitrate of silver and opium, immediately after the abstraction of a large quantity of blood, and those circumstances that led me to the use of the remedy.

In 1825, while in Canada, an epidemic catarrh prevailed amongst the inhabitants : in many of these cases, the bronchial affections were secondary to an ophthalmic attack, in which the conjunctiva was principally engaged. All the private practitioners used the usual mode of fomentation, blistering, and bleeding, without being successful. I alone had recourse, from the first commencement of the symptoms, to the use of nitrate of silver as an external application, with the happiest effects; and to this practice I was led by the opinion of my much respected friend, Dr. James Forbes. Purgatives and cupping were seldom required. From the magic-like effect of this remedy in dispelling the blood from the conjunctiva,—from the similarity of the catarrhal disposition noticed in this epidemic to something of the same kind of catarrhal epidemic in the West India fevers, where the gastric symptoms were most prominent, the idea struck me that the nitrate of silver, in conjunction with opium, might act powerfully in allaying the stomach affections ; and with this intention it was given in the case of Mr. Cotton, clerk of the market at Dominica, long after the appearance of black vomit, and most certainly with the effect of stopping the vomiting for eight or ten hours before death.

Emboldened by the result, I proposed the remedy, in the case of an artillery man named Ewing, to Mr. M'Gibbon, assistant surgeon of the 35th, under whose immediate care the man was.

The plan of treatment was commenced under the following circumstances :—

A man of the name of Sweeny, belonging to the same corps, had been sent to hospital from the same barrack thirty-six hours before Ewing: this man having died perfectly yellow, and with the symptoms of black vomit, in twenty-four hours after admission, and his body opened in twelve hours after, we came from the dissection of Sweeny to the examination of the fluid which Ewing was throwing up; and, upon the comparison of the fluid taken from Sweeny's stomach with that ejected by Ewing, the identity was beyond the possibility of a doubt, and, under these impressions, all hopes of his recovery was in our minds out of the question. It was under these circumstances that I proposed that as much blood as could be abstracted should be removed, and that we should then administer nitrate of silver and opium.

Blood to the extent of about thirty-two ounces was forced from the arm; but, after the arm was tied up, and whilst we were abstracting blood, the vomiting recurred. In the mean time, the pills with nitrate of silver and opium were prepared, and administered; and from the first dose the vomiting ceased. They were continued for six or eight hours; and a dose of croton oil was then administered. He was convalescent the third or fourth day, and had no return of fever; although he continued languid for some weeks.

It will be seen that the nitrate of silver, in the foregoing case, was administered for the purpose of obtaining its stimulant and tonic powers solely, although it may have acted beneficially by its power of decomposing muriatic acid.

